# Effective biosynthesis of 2,5-furandicarboxylic acid from 5-hydroxymethylfurfural via a bi-enzymatic cascade system using bacterial laccase and fungal alcohol oxidase

**DOI:** 10.1186/s13068-023-02406-z

**Published:** 2023-11-01

**Authors:** Fan Yang, Jiashu Liu, Bianxia Li, Huanan Li, Zhengbing Jiang

**Affiliations:** 1 State Key Laboratory of Biocatalysis and Enzyme Engineering, Hubei University, Wuhan, 430062 People’s Republic of China; 2 School of Life Science, Hubei University, Wuhan, 430062 People’s Republic of China

**Keywords:** Bacterial laccase, Alcohol oxidase, Enzymatic cascade, Biocatalysis, 2,5-Furandicarboxylic acid

## Abstract

**Background:**

As a cost-effective and eco-friendly approach, biocatalysis has great potential for the transformation of 5-hydroxymethylfurfural (HMF) into 2,5-furandicarboxylic acid (FDCA). However, the compatibility of each enzyme in the cascade reaction limits the transformation efficiency of HMF to FDCA.

**Results:**

Coupled with an alcohol oxidase from *Colletotrichum gloeosporioides* (*Cgl*AlcOx), this study aims to study the potential of bacterial laccase from *Bacillus pumilus* (*Bp*Lac) in an enzymatic cascade for 2,5-furandicarboxylic acid (FDCA) biosynthesis from 5-hydroxymethylfurfural (HMF). *Bp*Lac showed 100% selectivity for HMF oxidation and generated 5-hydroxymethyl-2-furancarboxylic acid (HMFCA). *Cgl*AlcOx was capable of oxidizing HMFCA to 2-formyl-5-furancarboxylic acid (FFCA). Both *Bp*Lac and *Cgl*AlcOx could oxidize FFCA to FDCA. At the 5 mM scale, a complete transformation of HMF with a 97.5% yield of FDCA was achieved by coupling *Bp*Lac with *Cgl*AlcOx in the cascade reaction. The FDCA productivity in the reaction was 5.3 mg/L/h. Notably, *Bp*Lac could alleviate the inhibitory effect of FFCA on *Cgl*AlcOx activity and boost the transformation efficiency of HMF to FDCA. Moreover, the reaction was scaled up to 40 times the volume, and FDCA titer reached 2.6 mM with a yield of 58.77% at 168 h.

**Conclusions:**

This work provides a candidate and novel insight for better design of an enzymatic cascade in FDCA production.

**Supplementary Information:**

The online version contains supplementary material available at 10.1186/s13068-023-02406-z.

## Background

2,5-Furandicarboxylic acid (FDCA) is considered one of the “Top 12” promising biomass-based building blocks [1]. Compared with petroleum-based polyethylene terephthalate (PET), biomass-based polyethylene furandicarboxylate (PEF) derived from FDCA not only exhibits excellent thermochemical properties with biodegradability, but also excellent gas barrier performance, recyclability, and extended mechanical properties [2]. FDCA production is generally initiated by the oxidation of 5-Hydroxymethylfurfural (HMF) [3, 4]. HMF is a promising chemical that can be transformed into various high value-added chemicals, such as furan and furan derivatives, carboxylic acids, lactones and lactam, diol and alkanes [5, 6], HMF can be produced from various C6 sugars, including glucose, fructose, sucrose, maltose, cellobiose, inulin, and starch [5]. In particular, the hydrolysis of cellulose from lignocellulosic biomass generates sugars, which can be used for HMF production after dehydration under acidic conditions [3, 7]. The formation of FDCA from HMF is divided into two pathways during the initial oxidation of HMF: (i) the alcohol group of HMF is oxidized to an aldehyde group and forms 2,5-diformylfuran (DFF); (ii) the aldehyde group of HMF is oxidized to a carboxyl group to generate 5-hydroxymethyl-2-furancarboxylic acid (HMFCA) [4]. Thereafter, 2-formyl-5-furancarboxylic acid (FFCA) is produced by oxidizing the aldehyde groups of DFF and the alcohol group of HMFCA, which can be further oxidized to form FDCA [4]. The traditional chemical approaches for FDCA synthesis from HMF can reach a sufficiently large industrial scale, while the long processing time, high temperatures, and noble metal catalyst requirements make them uneconomic [8, 9]. With regard to the increasing concern about environmental and political issues, cost-effective and eco-friendly biocatalysis is considered an alternative for chemical approaches in FDCA production.

Biocatalytic oxidation of HMF to FDCA can be achieved using either whole-cell or enzymatic conversion approaches under mild conditions [10–12]. Relatively high titers of FDCA are obtained via whole-cell oxidation using engineered strains. However, complex genetic manipulation and additional purification processes are needed because wild-type strains exhibit low conversion efficiency and other compounds present in the reaction mixture [10, 13]. To overcome this issue, enzymatic cascade strategies are proposed that can improve the efficiency of FDCA production from HMF [4]. In the Carbohydrate-Active enZYmes (CAZy) Database, Auxiliary Activity Family 5 (AA5) is divided into subfamily 1 (AA5_1) and subfamily 2 (AA5_2). AA5_1 contains glyoxal oxidases that can catalyze the oxidation of aldehydes to carboxylic acids with the reduction of O_2_ to H_2_O_2_ [14]. The enzymes that belong to AA5_2 are capable of oxidizing the alcohol group of various substrates into the corresponding aldehydes, such as primary alcohols and galactose [15]. The recently discovered fungi-derived alcohol oxidases (AlcOx, EC 1.1.3.13) belong to the AA5_2 subfamily and show broad substrate specificity [14, 16–19]. These enzymes have been widely used for FDCA production in enzymatic cascade reactions using HMF as the substrate [8, 10, 17, 20]. However, the catalytic mechanism of alcohol oxidase narrows the options of enzymes potentially involved in FDCA synthesis because many enzymes may be intolerant to H_2_O_2_. Therefore, widening the spectrum of oxidase is necessary to improve the compatibility of each enzyme in cascade reactions for FDCA synthesis.

Laccase (EC 1.10.3.2) belongs to multi-copper oxidase that can catalyze the oxidation of diverse phenolic and non-phenolic substrates along with the reduction of molecular oxygen to water [21]. The wide distribution of laccases in higher plants, fungi, bacteria, and insects brings different biological functions in nature [22]. Meanwhile, laccase is considered one of the most important biocatalysts for many industrial applications, such as green synthesis, bioremediation, and biosensors [23, 24]. For instance, fungal laccase is able to oxidize some primary alcohols to the corresponding carboxylic acids or aldehydes, which provides a promising route in green synthesis [25]. The redox mediator is required during the transformation of HMF to FDCA by fungal laccase in the laccase-mediator system (LMS) [26]. However, many synthetic mediators are potentially toxic and expensive [27]. CotA laccase from *Bacillus* species is well studied that can be used in many industrial applications. The CotA is located in the outer coat layer and consists of one of the key components of endospores [28]. Based on this characteristic, CotA has been proven to be responsible for providing resistance against chemicals and physical agents, such as H_2_O_2_, heat, and UV light [28, 29]. H_2_O_2_ is a typical inhibitor of enzymatic activity. Nevertheless, bacterial laccase from *Bacillus altitudinis* exhibits a much higher H_2_O_2_ tolerance than fungal laccases [30]. Also, we found that a bacterial laccase from *Bacillus pumilus* ZB1 (*Bp*Lac) can tolerate alkaline pH and degrade environmental pollutants effectively [24, 31]. Bacterial laccase shows better adaptivity to harsh industrial conditions than fungal laccase [32]. Thus, we speculate that the CotA laccase may be suitable for FDCA synthesis from HMF by cascade reactions together with alcohol oxidase. However, the conversion of HMF to FDCA by bacterial laccase in enzymatic cascade has not been well studied.

Herein, we aimed to exploit the potential of *Bp*Lac in FDCA synthesis using HMF as the precursor. A previously reported alcohol oxidase from *Colletotrichum gloeosporioides* was also employed in this study [16]. First, we assess the capacity of *Bp*Lac or alcohol oxidase for the oxidation of HMF, DFF, HMFCA, and FFCA. Second, we designed the enzymatic cascade reaction for FDCA synthesis from HMF using the two oxidases. Moreover, we explore the binding pattern of enzymes to the substrates in the bi-enzymatic cascade system via molecular docking. At last, the efficiency of the bi-enzymatic cascade system for FDCA synthesis on the scale-up process was investigated.

## Results and discussion

### Biotransformation of HMF and its oxidized derivatives by *Bp*Lac

**Fig. 1:**
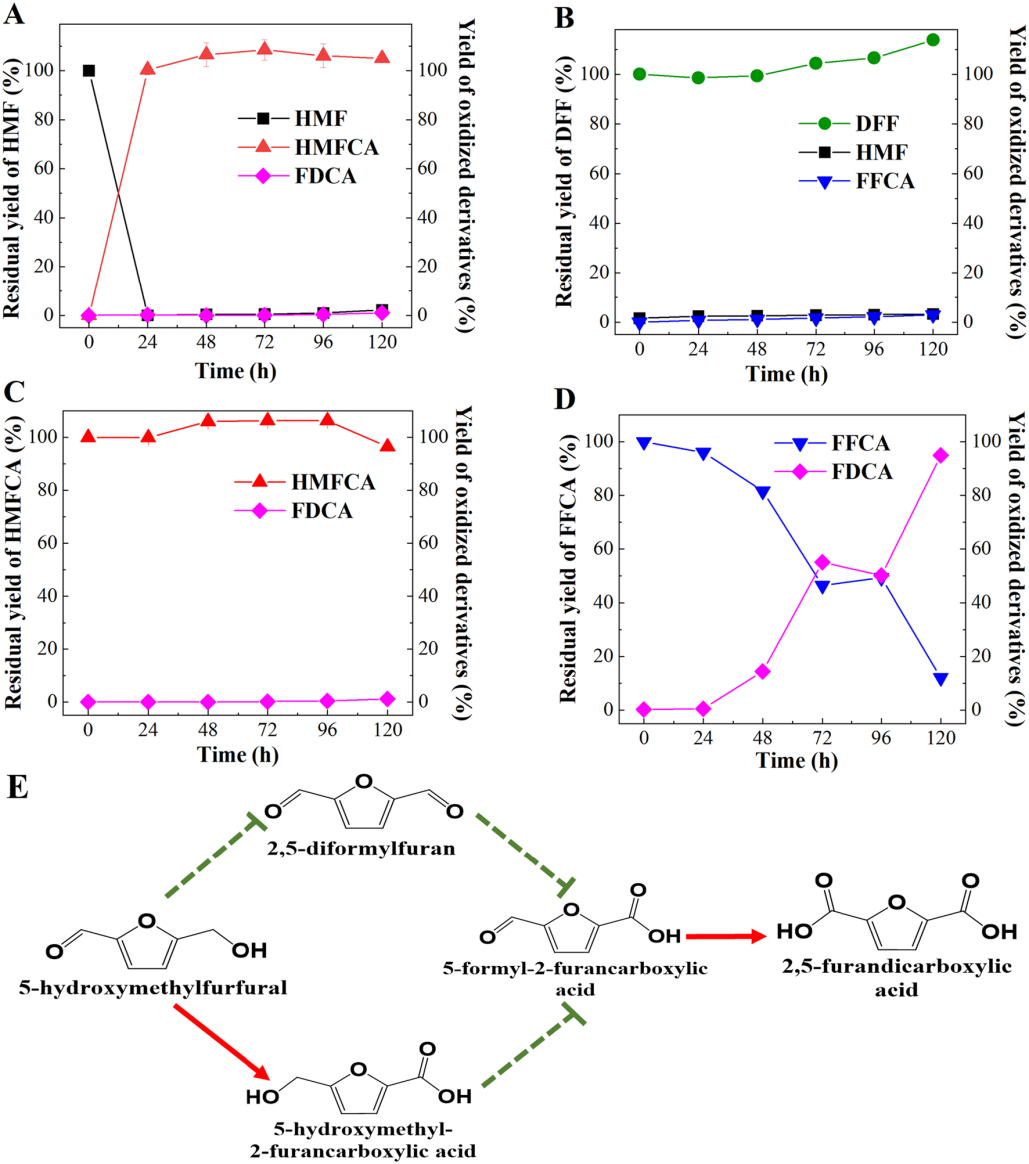
Analysis of the oxidation capacity of *Bp*Lac towards HMF and its oxidized derivatives. Time-course analysis of the oxidation potential of *Bp*Lac to **A** 5 mM HMF, **B** 5 mM DFF, **C** 5 mM HMFCA, and **D** 5 mM FFCA, respectively. **E** Schematic illustration of the catalyzing potential of *Bp*Lac to HMF and its oxidized derivatives.  means the reaction could not proceed,  means the reaction could proceed successfully

The purified *Bp*Lac was obtained through Ni-chelating affinity chromatography. The purity and the molecular weight of *Bp*Lac were confirmed via SDS‒PAGE (Additional file 1: Figure S2). The catalytic potential of *Bp*Lac toward HMF and its oxidized derivatives was investigated. As shown in Fig. 1, *Bp*Lac exhibited good catalytic performance for the oxidation of HMF and FFCA. When the concentration of HMF was 5 mM, HMF was completely converted to HMFCA at 100% selectivity after being treated by *Bp*Lac for 24 h (Fig. 1A). When DFF was used as the substrate in the reaction, the yield of FFCA reached only 2.9% after being treated by *Bp*Lac for 120 h (Fig. 1B). Similarly, when HMFCA was used as the substrate, no FFCA was detected and the yield of FDCA reached only 1.17% after being treated by *Bp*Lac for 120 h (Fig. 1C). We speculated that the oxidation of HMFCA to FFCA occurred spontaneously. The trace of FFCA might further transformed into FDCA by *Bp*Lac transiently. Notably, *Bp*Lac showed a good capacity for FDCA synthesis using 5 mM FFCA as the substrate (Fig. 1D). After being treated by *Bp*Lac for 120 h, the titer and yield of FDCA reached 4.69 mM with a yield of 94.94%.

To date, both bacterial laccases and fungal laccases show the potential in FDCA synthesis by oxidizing HMF and its derivatives [26, 33–35]. Laccases from bacteria generally have low redox potential (340–470 mV), while fungal laccases show high redox potential (490–790 mV) [36]. Relatively low redox potential limits the catalytic activity of bacterial laccases for many substrates. Previous study reveals that the redox mediators can enhance the capacity of laccases for oxidizing various substrates, including HMF [26, 27, 29, 37]. The typical mediator among the synthetic mediators, 2,2,6,6-tetramethyl-1-piperidinyloxy (TEMPO), is widely used in LMS for HMF oxidation by bacterial laccase [34, 35]. Nevertheless, the synthetic mediators are generally expensive and toxic [27]. In our study, without the redox mediators, *Bp*Lac was capable of oxidizing HMF to HMFCA with high selectivity and also oxidizing FFCA effectively. This characteristic of *Bp*Lac not only reduces the potential cost and health risk during HMF oxidation but may also reduce by-product formation in cascade reactions.

### Biotransformation of HMF and its oxidized derivatives by *Cgl*AlcOx

**Fig. 2:**
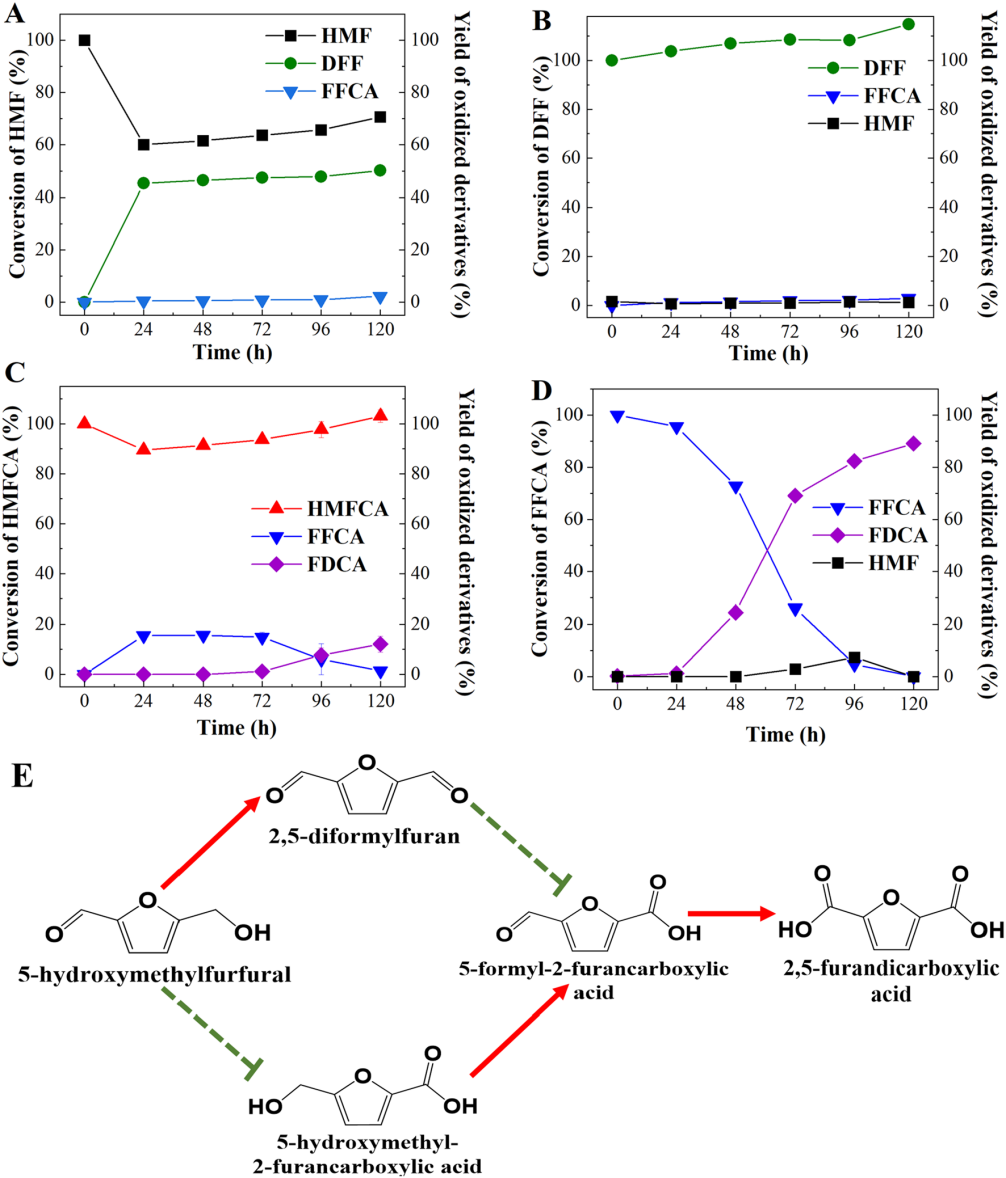
Analysis of the oxidation capacity of *Cgl*AlcOx towards HMF and its oxidized derivatives. Time-course analysis of the catalyzing capacity of *Cgl*AlcOx to each substrate, including **A** 5 mM HMF, **B** 5 mM DFF, **C** 5 mM HMFCA, and **D** 5 mM FFCA, respectively. **E** Schematic illustration of the catalyzing potential of *Cgl*AlcOx to HMF and its oxidized derivatives.  means the reaction could not proceed,  means the reaction could proceed successfully

Cleveland and co-workers characterized *Cgl*AlcOx systematically and considered it an alcohol oxidase [16]. They also found that *Cgl*AlcOx can completely oxidize HMF to form 91% of FFCA and 9% of FDCA in the presence of catalase and horseradish peroxidase [16]. The potential of *Cgl*AlcOx on HMF oxidation without the addition of other enzymes is unknown. Here, we aimed to assess the catalytic activity of *Cgl*AlcOx for the oxidation of HMF and its derivatives. The purified *Cgl*AlcOx was obtained through heterologous expression using *P. pastoris* X33 as the host. As expected, the molecular weight of recombinant *Cgl*AlcOx was 51.3 kDa, which was confirmed using SDS‒PAGE analysis (Additional file 1: Fig. S2). *Cgl*AlcOx showed a different selectivity in HMF oxidation compared with *Bp*Lac. When 5 mM of HMF served as the substrate, *Cgl*AlcOx catalyzed HMF to form DFF. The DFF yield reached 45.35% rapidly within 24 h and the final yield of DFF reached 50.14% after being treated by *Cgl*AlcOx for 120 h (Fig. 2A). *Cgl*AlcOx showed 100% selectivity for HMF oxidation to DFF because no HMFCA was detected in the reaction. As shown in Fig. 2B, when DFF was used as the substrate in the reaction, the yield of FFCA reached only 2.91% after being treated by *Cgl*AlcOx for 120 h. *Cgl*AlcOx was able to oxidize HMFCA and then generated FFCA and FDCA (Fig. 2C). With the decrease in HMFCA, the yield of the intermediate FFCA reached 15.57% at 24 h. After the reaction proceeded for 72 h, the final product FDCA appeared along with the disappearance of the intermediate FFCA. The yield of FDCA reached 12.07% with respect to HMFCA after being treated by *Cgl*AlcOx for 120 h. As shown in Fig. 2D, when FFCA served as the substrate, the targeted product FDCA formed effectively after being treated by *Cgl*AlcOx for 120 h. The yield of FDCA reached approximately 89.12% after the reaction proceeded for 120 h.

As previous literature mentioned, *Cgl*AlcOx is classified as a member of AA5_2 subfamily and exhibits high specific activities toward a wide range of substrates, such as diols, primary alcohols, and aryl alcohols [16]. The results presented in this study were slightly different from those of previous research. In the previous study, *Cgl*AlcOx was capable of oxidizing HMF and HMFCA while being disabled to oxidize DFF and FFCA [16]. In the present study, we found that *Cgl*AlcOx could oxidize HMF, HMFCA, and FFCA, but not DFF. Therefore, *Cgl*AlcOx not only showed a potential to catalyze HMFCA to FFCA but also transformed FFCA to FDCA effectively, implying that *Cgl*AlcOx can be involved in the last two steps of the oxidation of HMF to FDCA.

### Establishment of the enzymatic cascade reaction for FDCA synthesis by *Bp*Lac and *Cgl*AlcOx

**Fig. 3:**
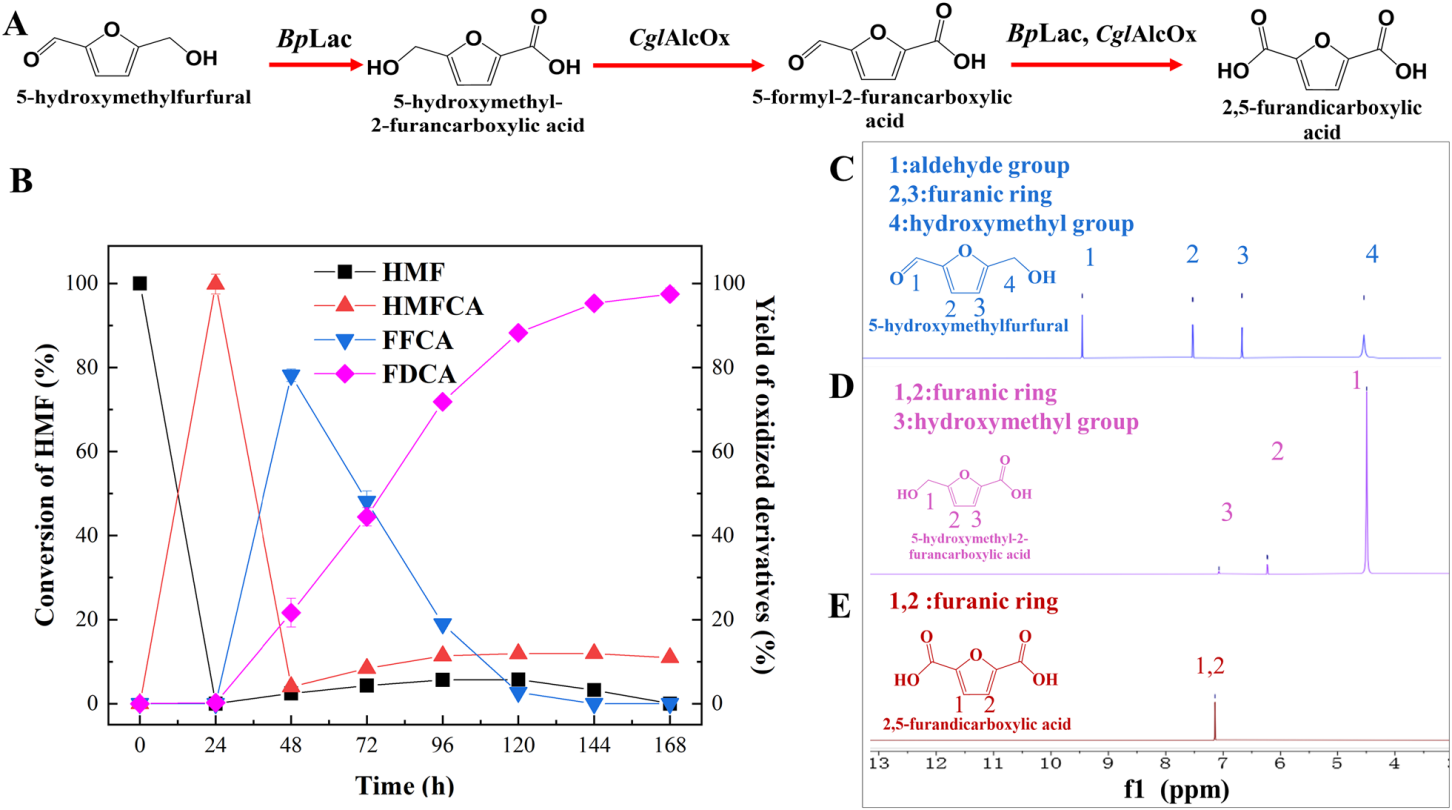
The enzymatic cascade reaction for FDCA biosynthesis in the present study. **A** Schematic representation of the proposed enzymatic cascade reaction for FDCA synthesis from HMF using *Bp*Lac and *Cgl*AlcOx. **B** Analysis of the FDCA synthesis catalyzed by *Bp*Lac and *Cgl*AlcOx using HMF as the substrate. *Bp*Lac was initially added to the reactions at 0 h. *Cgl*AlcOx was added into the reactions at 24 h when the HMF was completely transformed into HMFCA by *Bp*Lac. **C** ^1^H-NMR spectra for initial HMF in the reaction mixture. **D** ^1^H-NMR spectra for the complete formation of HMFCA from HMF catalyzed by *Bp*Lac. **E** ^1^H-NMR spectra for identification of the final product FDCA in the enzymatic cascade.  means the reaction could proceed successfully

As we described above, neither *Bp*Lac nor *Cgl*AlcOx could convert DFF to FFCA. Although *Cgl*AlcOx is capable of oxidizing HMFCA and FFCA, the selective oxidation of HMF by *Cgl*AlcOx to form DFF limited the accumulation of HMFCA at the first step. *Bp*Lac is well suited to overcome this issue because HMF could be selectively oxidized to HMFCA with 100% selectivity. Thus, the enzymatic cascade reaction for FDCA synthesis by *Bp*Lac and *Cgl*AlcOx was established and divided into two steps. First, we added only *Bp*Lac into the reaction using HMF as the substrate. After HMF was completely transformed into HMFCA by *Bp*Lac, the second step was initiated by adding *Cgl*AlcOx into the reaction for HMFCA oxidation. Then the intermediate FFCA could be transformed into FDCA with the help of both *Bp*Lac and *Cgl*AlcOx (Fig. 3A). As shown in Fig. 3B, the conversion of HMF to FDCA on a 5 mM scale was achieved with good yield. When the reaction proceeded at first step, HMF was completely transformed to HMFCA within 24 h. Thereafter, *Cgl*AlcOx was supplemented to the reaction. The HMFCA amount decreased to 4.02% along with the FFCA amount increased to 78.14% at 48 h, indicating that *Cgl*AlcOx could effectively convert HMFCA to FFCA in the cascade reaction. The yield of FDCA increased constantly along with the decrease of FFCA when the reaction proceeded after 48 h. After the reaction continuously proceeded for 168 h, the FDCA titer reached 4.88 mM with a yield of 97.5%. The FDCA productivity in the reaction was 5.3 mg/L/h. Additionally, for the characteristic signals of each chemical, the disappearance of HMF and the formation of HMFCA were observed when *Bp*Lac completely converted HMF to HMFCA. When *Cgl*AlcOx was added to the reaction mixtures, the intermediate HMFCA was rapidly transformed into FDCA. The ^1^H-NMR analysis confirmed that only the characteristic signal of FDCA was detected in the final product (Fig. 3D–E). Therefore, ^1^H-NMR spectra confirmed that HMF could be almost completely transformed into FDCA using enzymatic cascade that was established in our study.

Compared with fungal laccases, bacterial laccases recently show great potential in cascade reactions for FDCA synthesis. Chang and co-workers designed a tandem biocatalysis strategy using immobilized laccase from *Bacillus subtilis* TJ-102 and Novozym 435 (immobilized lipase B from *Candida Antarctica*), which provides a 94.2% FDCA yield from HMF [38]. Coupling with catalase, TEMPO, and H_2_O_2_, laccase from *B. subtilis* 168 can effectively utilize HMF as the substrate and yield 97.1% of FDCA [35]. As the above literature reported, the redox mediator, TEMPO, is used in enzymatic cascade reactions. Compared with the studies that added the mediators, we found that the complete transformation of HMF with a 97.5% yield of FDCA was achieved by coupling laccase from *B. pumilus* ZB1 and only alcohol oxidase. A comparison of the results of different enzymatic cascades is presented in Additional file 1: Table S2. An increase in substrate HMF concentration and higher reaction efficiency for FDCA synthesis are desired in enzymatic cascade reactions. Notably, most reported cascades generally need three or more enzymes to catalyze HMF into FDCA. In our study, two oxidases catalyzed HMF to FDCA with high efficiency, implying that reducing the enzyme dosage might lower the cost during the biosynthesis of FDCA. Previous studies generally establish the enzymatic cascade reactions by eliminating the generated H_2_O_2_ from alcohol oxidase catalysis [10, 17]. In the present work, the transformation efficiency in cascade reaction is much higher than that of the single reactions, implying that laccase from *B. pumilus* ZB1 may be tolerant H_2_O_2_ generated by *Cgl*AlcOx oxidation. Notably, *Cgl*AlcOx showed much higher oxidation capacity toward HMFCA in enzymatic cascade reaction compared with its single reaction. Here, we speculated that the intermediate FFCA and final product FDCA might inhibit the catalytic activity of *Cgl*AlcOx toward HMFCA. Bacterial laccase might alleviate the inhibitory effect of FFCA and FDCA on *Cgl*AlcOx.

### Analysis of the inhibitory effect of FFCA and FDCA on *Cgl*AlcOx activity

**Fig. 4:**
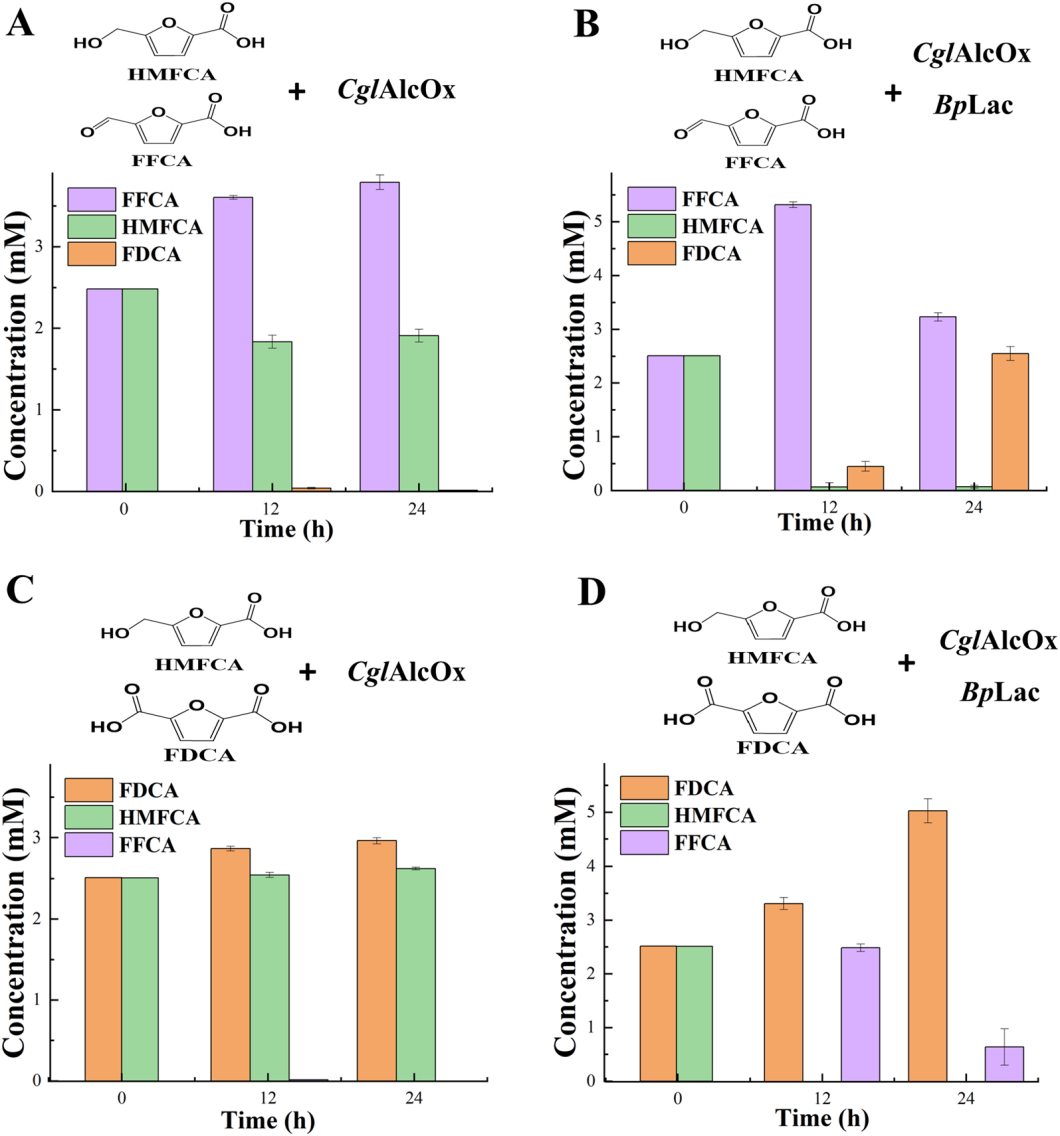
Analysis of the potential inhibitory effect of FFCA/FDCA on *Cgl*AlcOx activity for HMFCA oxidation. **A** Measurement of the oxidation capacity of *Cgl*AlcOx using mixed substrates containing 2.5 mM FFCA and 2.5 mM HMFCA. **B** Verification of the role of *Bp*Lac in alleviating the inhibitory effect of FFCA on *Cgl*AlcOx in cascade reactions containing 2.5 mM FFCA and 2.5 mM HMFCA **C**. Measurement of the oxidation capacity of *Cgl*AlcOx using mixed substrates containing 2.5 mM FDCA and 2.5 mM HMFCA. **D** Analysis of the oxidation capacity of mixed enzymes (*Bp*Lac and *Cgl*AlcOx) to the mixed chemicals (2.5 mM HMFCA and 2.5 mM FDCA)

To confirm whether FFCA and FDCA could inhibit *Cgl*AlcOx activity during the oxidation of HMFCA, we mixed the intermediate FFCA or the product FDCA to the reaction mixtures containing HMFCA and *Cgl*AlcOx, respectively. FFCA might have an inhibitory effect on *Cgl*AlcOx activity during HMFCA oxidation (Fig. 4A). When the reaction mixture contained both HMFCA and FFCA, the amount of FFCA increased to 3.6 mM within 12 h while only a trace amount of FDCA was detected. The amount of HMFCA was stable when the reaction proceeded for 24 h. Interestingly, when the reaction mixture contained *Bp*Lac, HMFCA was almost completely transformed into FFCA within 12 h (Fig. 4B). After being treated with *Cgl*AlcOx and *Bp*Lac for 24 h, FFCA amount decreased to 3.23 mM and FDCA amount increased to 2.55 mM.

As shown in Fig. 4C, FDCA probably also inhibited *Cgl*AlcOx activity for HMFCA oxidation due to HMFCA amount had no obvious change after the reaction proceeded for 24 h. However, when the reaction mixture containing HMFCA, FDCA, *Cgl*AlcOx, and *Bp*Lac, the disappearance of HMFCA and the formation of FFCA have occurred simultaneously within 12 h (Fig. 4D). This result revealed that the presence of FDCA would not inhibit HMFCA oxidation by *Cgl*AlcOx. The continuously decreasing FFCA of 0.64 mM and increasing FDCA of 5.03 mM were detected at 24 h. This result also confirmed that the presence of *Bp*Lac in the reaction could improve the transform efficiency of HMFCA by *Cgl*AlcOx. In our study, we found that the FFCA has an inhibitory effect on alcohol oxidase activity toward HMFCA. Interestingly, *Bp*Lac could alleviate the inhibitory effect of FFCA on *Cgl*AlcOx, and boost the efficiency of the enzymatic cascade for FDCA synthesis. H_2_O_2_ generated by alcohol oxidase is the limiting factor to enzymatic activity during cascade reactions [8]. *Bp*Lac might have a good tolerance towards H_2_O_2_ generated from *Cgl*AlcOx catalysis. Therefore, *Bp*Lac is suitable for the high-efficiency production of FDCA from HMF by coupling with *Cgl*AlcOx.

Additionally, alleviating the inhibitory effect could be achieved by increasing the relative amount of *Cgl*AlcOx in the reaction mixtures. Different from the original reaction mixtures containing 5 mM HMFCA and 1 U/mL *Cgl*AlcOx, lowering the amount of HMFCA to 1 mM or increasing the *Cgl*AlcOx loading to 5 U/mL benefit the transformation of HMFCA proceeded effectively (Additional file 1: Fig. S3). These results provided a potential strategy that may improve the efficiency of FDCA biosynthesis in future studies.

### Prediction of the key residues of *Cgl*AlcOx and *Bp*Lac for binding substrates via molecular docking

**Fig. 5:**
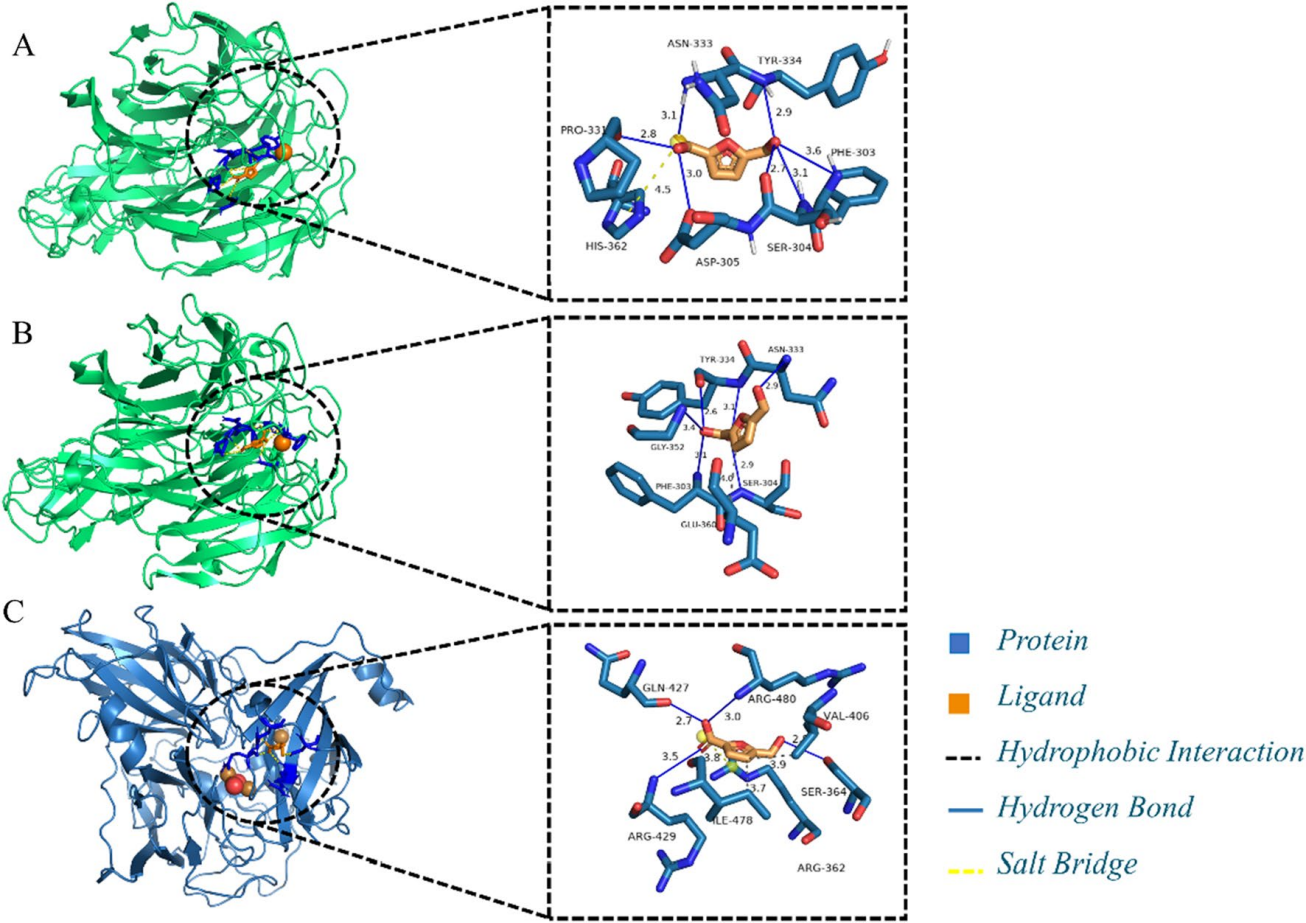
Representation of molecular docking results of the enzyme–substrate complex. **A** *Cgl*AlcOx-HMFCA. **B** *Cgl*AlcOx-FFCA. **C** *Bp*Lac-FFCA

To deeply understand the enzymatic cascade system for FDCA synthesis, we investigated the biotransformation of HMFCA and FFCA by *Cgl*AlcOx via molecular docking, as well as the biotransformation of FFCA by *Bp*Lac. The prediction of the binding site between enzyme and substrate is presented in Fig. 5. Subsequently, the molecular interaction of the enzyme–substrate complex was analyzed in detail (Table 1). *Cgl*AlcOx-HMFCA has a docking score of − 6.34 kcal/mol which formed six hydrogen bonds with Phe303, Ser304, Asp305, Pro331, Asn333, and Tyr334, and one salt bridge with His362. *Cgl*AlcOx-FFCA has a docking score of − 5.52 kcal/mol which formed five hydrogen bonds with Phe303, Ser304, Asn333, Tyr334, and Gly352, and one hydrophobic interaction with Glu360. *Bp*Lac formed four hydrogen bonds (Ser364, Gln427, Arg429, and Arg480), two hydrophobic interactions (Val406 and Ile478), and one salt bridge (Arg362) with FFCA, which resulted in the minimum binding energy of − 4.63 kcal/mol. The lowest binding energy represents highly stable conformation of substrate to enzyme [28]. In our study, both HMFCA and FFCA could form stable complex with *Cgl*AlcOx. Four amino acid residues, including Phe303, Ser304, Asn333, and Tyr334, are involved in binding to both HMFCA and FFCA. *Cgl*AlcOx showed a salt bridge with HMFCA, while forming one hydrophobic interaction with FFCA. The hydrogen bonds and hydrophobic interactions contribute to the stability of enzyme–substrate complex [39]. Compared with hydrogen bonds and hydrophobic interactions, the salt bridge has proven to be the strongest interaction between enzyme and substrate [40]. Therefore, the formation of one salt bridge with His362 may contribute to lower binding energy of HMFCA to *Cgl*AlcOx. In addition, arginine in the substrate binding pocket of bacterial laccase is considered to be important for substrate oxidation [41]. In the present study, three arginine residues from the active site of *Bp*Lac were found to be involved in binding to FFCA. Thus, the presence of *Bp*Lac in the cascade reaction may reduce the binding of FFCA to *Cgl*AlcOx and then oxidize FFCA to FDCA.

**Table 1:** Molecular docking analysis of the enzyme–substrate complex and the key amino acid residues

Enzyme–substrate complex	Binding amino acid residues			Binding energy (kcal/mol)
	Hydrogen bonds	Hydrophobic interactions	Salt bridges	
*Cgl*AlcOx-HMFCA	Phe303, Ser304, Asp305, Pro331, Asn333, Tyr334		His362	− 6.34
*Cgl*AlcOx-FFCA	Phe303, Ser304, Asn333, Tyr334, Gly352	Glu360		− 5.52
*Bp*Lac-FFCA	Ser364, Gln427, Arg429, Arg480	Val406, Ile478	Arg362	− 4.63

### Scale-up experiment

**Fig. 6:**
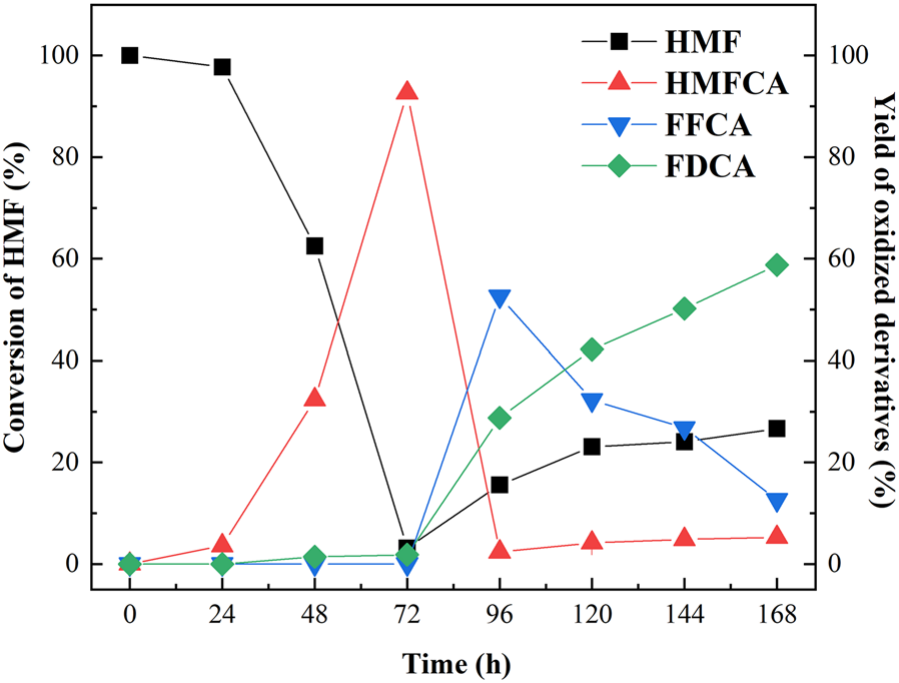
Time-course analysis of FDCA biosynthesis using bi-enzymatic cascade system at 0.2 L scale-up reaction

The enzymatic cascade reaction was scaled up to 0.2 L in a 2 L flask. As shown in Fig. 6, the bi-enzymatic cascade system was successfully scaled up to 40 times the volume. With HMF as the substrate, the yield of HMFCA reached 92.45% after being treated by *Bp*Lac for 72 h. After *Cgl*AlcOx was added and the reaction proceeded for 96 h, the yield of FFCA and FDCA reached 52.59% and 28.75%, respectively. When the cascade reaction proceeded for 168 h, FDCA titer reached 2.6 mM with a yield of 58.77%. Scale-up brings the challenge to the effective biotransformation of HMF to FDCA. In scale-up reaction, *Bp*Lac took a longer time for the complete oxidation of HMF. Compared with the 5 mL reaction system, the yield of FDCA in the scaled-up system decreased from 97.5% to 58.77%. Due to oxygen transfer and mixing may be negatively affected, the catalytic efficiency decrease in scale-up system is expected [42]. An improvement of the transformation efficiency of the bi-enzymatic cascade system in scale-up process is our effort in the future.

## Conclusions

The present study established an effective bi-enzymatic cascade system for FDCA synthesis using *Bp*Lac and *Cgl*AlcOx. These two oxidases played a complementary role in catalyzing HMF to form FDCA. *Bp*Lac showed good selectivity for the oxidation of HMF without the addition of redox mediators. *Bp*Lac could alleviate the inhibitory effect of FFCA on *Cgl*AlcOx activity and then boost the synthesis of FDCA in the cascade reaction. The key residues of *Bp*Lac and *Cgl*AlcOx that are involved in binding to the substrates were identified. The synthesis of FDCA in cascade reaction also succeeded when the volume scaled up to 0.2 L.

## Materials and methods

### Strains and chemicals

*Escherichia coli* DH5α competent cells were used for cloning plasmids and grown in Luria–Bertani (LB) medium. *Pichia pastoris* X33 was used for the production of heterologous protein. YPD, BMGY, and BMMY media were prepared according to the manual of the *Pichia* Expression Kit.

HMF, DFF, HMFCA, FFCA, and FDCA were purchased from Shanghai Aladdin Biochemical Technology Co., Ltd., China. 2,2′-Azino-bis (3-ethylbenzothiazoline-6-sulfonic acid) (ABTS) was purchased from Sigma‒Aldrich Co., Ltd., USA.

### Heterologous expression and purification

In the present study, the eukaryotic heterologous expression system was employed for the expression of laccase from *B. pumilus* ZB1 (GenBank: MW373470) and alcohol oxidase from *C. gloeosporioides* (GenBank: ELA25906). The laccase gene sequence was obtained in our previous study [24], and the alcohol oxidase gene sequence was obtained from the literature [16, 43]. The plasmid pET28a(+)-*lac* served as the template for laccase gene amplification using forward primer 5′-AAGAGAGGCTGAAGCTGAATTCATGAACCTAGAAAAATTTGTTG-3′ and reverse primer 5′-TGAGATGAGTTTTTGTTCTAGAGAAATAATATCCATCGGCCGCAT-3′. The signal peptide of alcohol oxidase was predicted by the SignalP 5.0 server (https://services.healthtech.dtu.dk/service.php?SignalP-5.0). The sequence of alcohol oxidase without its own signal peptide was synthesized according to the released data from the NCBI database. Both the laccase gene (*Bplac*) and alcohol oxidase gene (*CglAlcOx*) were cloned into linearized pPICZαA (digestion with *Eco*R I and *Xba* I). Thereafter, the recombinant plasmids pPICZαA-*Bplac* and pPICZαA-*CglAlcOx* were linearized with *Sac* I and transformed into *P. pastoris* X33 by the electroporation method. The positive transformants were selected from YPD plates supplemented with 100 μg/mL Zeocin.

The *P. pastoris* transformants X33/pPICZαA-*Bplac* and X33/pPICZαA-*CglAlcOx* were inoculated into 50 mL of YPD medium at 28 °C and shaken at 220 rpm overnight. The actively growing cultures [2% inoculum size (v/v)] were transferred into BMGY medium supplemented with 0.5 mM CuSO_4_ and 0.2% biotin. When the OD600 reached 2–6 after incubation for approximately 16–18 h, the yeast cells were transferred into BMMY medium supplemented with 0.5 mM CuSO_4_ and 0.2% biotin. The cultures were continuously incubated at 18 °C with shaking at 200 rpm. The induction of *Bp*Lac and *Cgl*AlcOx was proceeded by adding 0.5% and 1% methanol, respectively.

The cultures were harvested when *Bp*Lac and *Cgl*AlcOx showed their highest activities. The supernatants were concentrated by a Vivaflow 200 ultrafiltration system with a 10 kDa cut-off (Sartorius, Germany). For the purification of *Bp*Lac and *Cgl*AlcOx, Ni–NTA column was equilibrated with 50 mM sodium phosphate buffer at pH 8.0 and pH 7.5, respectively. The concentrated supernatants were loaded onto the column and eluted with a linear gradient of imidazole from 20 to 300 mM in 50 mM sodium phosphate buffer containing 0.5 M NaCl. The purified *Bp*Lac and *Cgl*AlcOx were analyzed via SDS‒PAGE.

### Enzymatic activity assay

Laccase activity was assayed at room temperature, with ABTS as the substrate in sodium acetate buffer. The reaction mixture contained 50 μL of 20 mM ABTS, 940 μL of 0.2 M sodium acetate buffer (pH 5.0), and 10 μL of purified *Bp*Lac. The oxidation of ABTS was monitored at 420 nm (*ε* = 36,000 M^−1^ cm^−1^) as previously reported [24]. The amount of enzyme oxidizing 1 μmol ABTS per minute is defined as one unit of enzyme activity.

Alcohol oxidase activity was measured at room temperature by determining ABTS oxidation at 420 nm. The reaction mixture consisted of 50 mM sodium phosphate buffer (pH 8.0), 0.46 mM ABTS, 30 U/mL Horseradish peroxidase (HRP), 25 mM veratryl alcohol, and purified *Cgl*AlcOx. The previous study mentioned that the oxidation of the alcohol group on substrates by alcohol oxidase consumes 1 equivalent of O_2_ and produces 1 equivalent of H_2_O_2_ [17]. The subsequent oxidation of ABTS by HRP consumes 2 equivalents of H_2_O_2_ [44]. Thus, one unit of *Cgl*AlcOx activity was defined as the amount of *Cgl*AlcOx required to oxidize 2 μmol ABTS per minute.

### Biotransformation of HMF and its derivatives

Biotransformation of HMF and its derivatives (DFF, HMFCA, and FFCA) by *Bp*Lac and *Cgl*AlcOx was performed. The reaction mixtures in a total volume of 5 mL contained 50 mM sodium phosphate buffer (pH 7.0), 5 mM individual substrate (HMF, DFF, HMFCA, and FFCA), and 1 U/mL purified enzyme (*Bp*Lac or *Cgl*AlcOx). Reactions were conducted at 28 °C with shaking at 220 rpm for 120 h. Samples were taken every 24 h, and the reactions were stopped by adjusting the pH to 2.0 through the addition of 6 M HCl. The transformed products were analyzed through HPLC. All experiments were performed in triplicate.

### HPLC analysis

HMF and its oxidized derivatives were analyzed using an Agilent 1260 series HPLC system (Agilent, Wald Bronn, Germany) equipped with a Bio-Rad Aminex HPX-87H Column and a UV detector (wavelength = 264 nm). H_2_SO_4_ (7.5 mM) was used as the mobile phase. The separation and identification of these chemicals was conducted at 60 °C with a flow rate of 0.6 mL/min. The injection volume was 20 μL. The retention times of HMF, HMFCA, DFF, FFCA and FDCA were 29.74, 20.47, 36.2, 22.42, and 16.15 min, respectively. The titer (mM) of the oxidized products was calculated based on the plotted standard curve. The yield (%) of the oxidized products was calculated using the following formula (1):

1 $$ {\text{Yield}}\left( \% \right) = \frac{{{\text{Oxidized product}}\left( {{\text{mM}}} \right)}}{{{\text{Substrate HMF}}\left( {{\text{mM}}} \right)}} \times 100\% $$

### Enzymatic cascade reaction

A two-step enzymatic cascade reaction for FDCA synthesis was established in our study. The cascade reaction was performed in a total volume of 5 mL containing 50 mM sodium phosphate buffer (pH 7.0), 5 mM HMF, 1 U/mL purified *Bp*Lac, and 1 U/mL purified *Cgl*AlcOx. In the first step, *Bp*Lac was added to the reaction mixture together with the substrate HMF. Reactions were performed at 28 °C with shaking at 220 rpm for 24 h. In the second step, *Cgl*AlcOx was added to the mixture, and the reaction was continued at 28 °C with shaking at 220 rpm. The samples were harvested from the cascade reaction every 24 h. The converted products were analyzed via HPLC as described above.

### ***Characterization of the enzymatic cascade by ***^***1***^***H-NMR***

^1^H-NMR was used to analyze the formation of FDCA from HMF oxidation using an enzymatic cascade in this study. The standard chemicals of HMF, DFF, HMFCA, FFCA, and FDCA were dissolved in D_2_O. The chemical shifts of each chemical were characterized using an Avance NEO 400 NMR spectrometer (Bruker, Germany). The ^1^H-NMR spectrum and the characteristic signals of each chemical are presented in Additional file 1: Figure S1 and Table S1, respectively. Consistent with a previous study [14], the monohydrated form of DFF was observed in the ^1^H-NMR profile of DFF. The samples of the enzymatic cascade reaction were collected at three different time points. First, the sample was obtained at the beginning of the reaction. Second, the sample was withdrawn from the reaction mixtures when the HMF was completely transformed to HMFCA by *Bp*Lac. Thereafter, the final transforming product was used to confirm whether FDCA was the main product in the mixture. The targeted chemicals were separated from enzymes using ultrafiltration (nominal molecular weight cut-off of 10 kDa). Then the samples were dissolved in 10% D_2_O (v/v) and used for ^1^H-NMR analysis.

### Analysis of the potential inhibitory effect of FFCA and FDCA on *Cgl*AlcOx activity toward HMFCA

Whether FFCA and FDCA could inhibit *Cgl*AlcOx activity was verified following four experiments. (i) Each 2.5 mM FFCA and 2.5 mM FDCA was added into the reaction mixtures that contained 2.5 mM HMFCA and 1 U/mL *Cgl*AlcOx. (ii) Each 2.5 mM FFCA and 2.5 mM FDCA was added into the reaction mixtures that contained 2.5 mM HMFCA, 1 U/mL *Bp*Lac, and 1 U/mL *Cgl*AlcOx. (iii) The reaction mixtures contained 1 U/mL *Cgl*AlcOx and 1 mM HMFCA. (iv) The reaction mixtures contained 5 U/mL *Cgl*AlcOx and 5 mM HMFCA. All the reaction mixtures contained 50 mM sodium phosphate buffer (pH 7.0) and were then incubated at 28 °C with shaking at 220 rpm. The yield of FDCA was measured using HPLC as described above. All experiments were carried out in triplicate.

### Homology modeling and molecular docking

The 3D structural models of *Bp*Lac and *Cgl*AlcOx were predicted using the SWISS-MODEL server (https://swissmodel.expasy.org/). The model of *Bp*Lac was obtained as we previously reported [24]. The model of *Cgl*AlcOx shared 87.14% sequence identity with (PDB ID: 5C92). The structures of HMFCA and FFCA were obtained from the PubChem database (https://pubchem.ncbi.nlm.nih.gov/). Molecular docking for *Cgl*AlcOx-HMFCA, *Cgl*AlcOx-HMFCA, and *Bp*Lac-FFCA was conducted using AutoDock 4.2 software. The Lamarckian genetic algorithm (LGA) was applied to the interaction pattern between enzymes and ligands with 100 independent genetic algorithm (GA) runs. All other docking parameters were set following our previous study [24]. The optimal docking results were evaluated through the minimum binding energy. Thereafter, the interaction pattern of the enzyme–substrate complex was analyzed using the Protein–Ligand Interaction Profiler (PLIP) server (https://plip-tool.biotec.tu-dresden.de/plip-web/plip/index) [45]. The results were visualized in PyMol 2.2.0 [46].

### Scale-up for FDCA synthesis using the bi-enzymatic cascade system

The scale-up reaction was conducted with the total volume of 0.2 L. The reaction was performed in a 2 L flask. The compositions in the reaction were 50 mM sodium phosphate buffer (pH 7.0), 5 mM HMF, 1 U/mL purified *Bp*Lac, and 1 U/mL purified *Cgl*AlcOx. The reaction was performed with a slight modification. Briefly, the first step of the reaction mixture containing *Bp*Lac and HMF proceeded at 28 °C with shaking at 220 rpm for 72 h. The second step was continuously proceeded for 96 h under the same conditions and initiated by adding *Cgl*AlcOx to the reaction. The products were analyzed via HPLC as described above.

### Supplementary Information

**Additional file 1: Figure S1.** ^1^H-NMR spectra of HMF and its oxidized derivatives. **Figure S2.** SDS–PAGE analysis of purified recombinant proteins. **Figure S3.** Verification of the product inhibition on HMFCA oxidation by *Cgl*AlcOx through two individual experiments. ** Table S1.** The chemical shifts of characteristic peaks of HMF and its derivatives. **Table S2.** Summary of previously reported FDCA production from HMF via enzymatic cascade reactions.

## Data Availability

All data generated or analyzed during this study are included in this published article and its supplementary information files.

## Abbreviations

HMF: 5-Hydroxymethylfurfural
DFF: 2,5-Diformylfuran
HMFCA: 5-Hydroxymethyl-2-furancarboxylic acid
FFCA: 2-Formyl-5-furancarboxylic acid
FDCA: 2,5-Furandicarboxylic acid
ABTS: 2,2ʹ-Azino-bis (3-ethylbenzothiazoline-6-sulfonic acid
HRP: Horseradish peroxidase
*Cgl*AlcOx: Alcohol oxidase from *Colletotrichum gloeosporioides*
*Bp*Lac: Bacterial laccase from *Bacillus pumilus*
LMS: Laccase-mediator system
TEMPO: 2,2,6,6-Tetramethyl-1-piperidinyloxy
CAZy: Carbohydrate-Active enZYmes
AA5: Auxiliary Activity Family 5
LGA: The Lamarckian genetic algorithm
GA: Genetic algorithm
PLIP: Protein–Ligand Interaction Profiler
