# Supplementary material for: Effective biosynthesis of 2,5-furandicarboxylic acid from 5-hydroxymethylfurfural via a bi-enzymatic cascade system using bacterial laccase and fungal alcohol oxidase
Source: Biotechnol Biofuels Bioprod. 2023 Nov 1;16:164. doi: 10.1186/s13068-023-02406-z (PMC10621202; doi:10.1186/s13068-023-02406-z)
Supplement: Supplementary file 1 — Additional file 1: Figure S1. 1H-NMR spectra of HMF and its oxidized derivatives. Figure S2. SDS–PAGE analysis of purified recombinant proteins. Figure S3. Verification of the product inhibition on HMFCA oxidation by CglAlcOx through two individual experiments. Table S1. The chemical shifts of characteristic peaks of HMF and its derivatives. Table S2. Summary of previously reported FDCA production from HMF via enzymatic cascade reactions. [file 13068_2023_2406_MOESM1_ESM.docx]

**Supplementary materials**

**Effective biosynthesis of 2,5-furandicarboxylic acid from 5-hydroxymethylfurfural via a bi-enzymatic cascade system using bacterial laccase and fungal alcohol oxidase**

Fan Yang^1, 2, #^, Jiashu Liu^1, 2, #^, Bianxia Li^1, 2^, Huanan Li^1, 2^, Zhengbing Jiang^1, 2*^

^1^*State Key Laboratory of Biocatalysis and Enzyme Engineering, Hubei University, Wuhan, 430062, P.R. China*

^2^*School of Life Science, Hubei University, Wuhan, 430062, P.R. China*

^*^Corresponding authors

Zhengbing Jiang

E-mail: Zhengbing Jiang

^#^F. Yang and J. Liu contributed equally to this work.


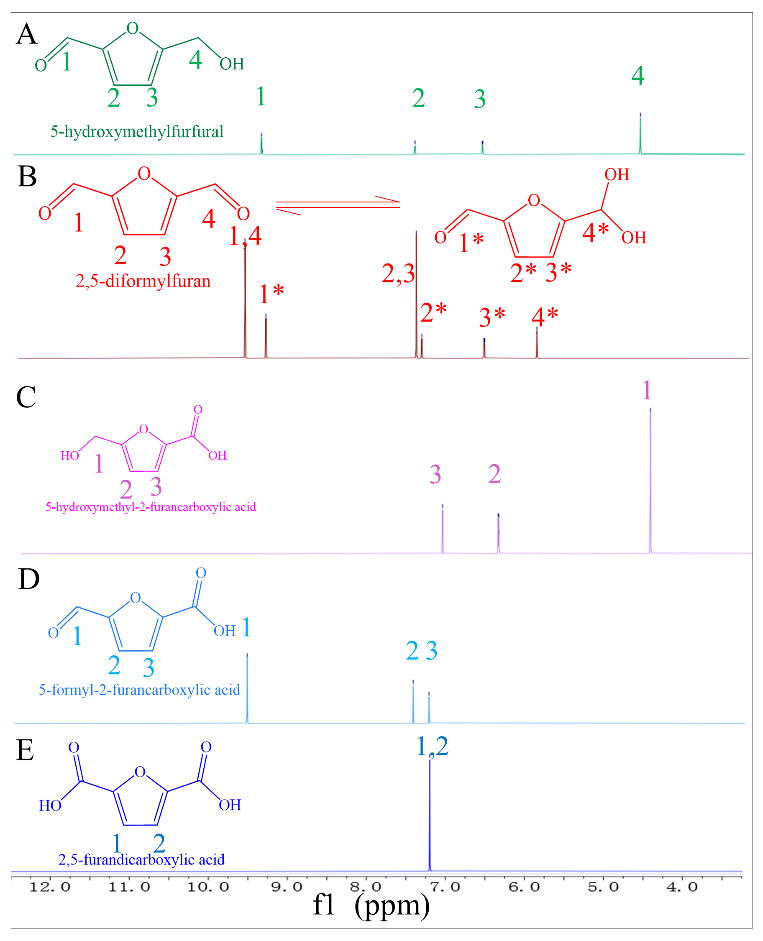


**Figure S1** ^1^H-NMR spectra of HMF and its oxidized derivatives.


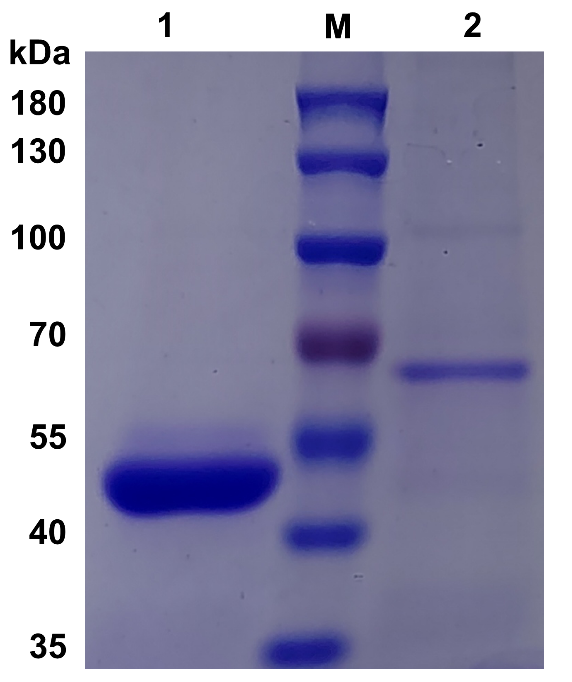


**Figure S2** SDS‒PAGE analysis of purified recombinant proteins. Analysis of purified recombinant proteins by 10% SDS-PAGE. Lane 1: *Cgl*AlcOx; lane 2: *Bp*Lac; lane M: protein marker.


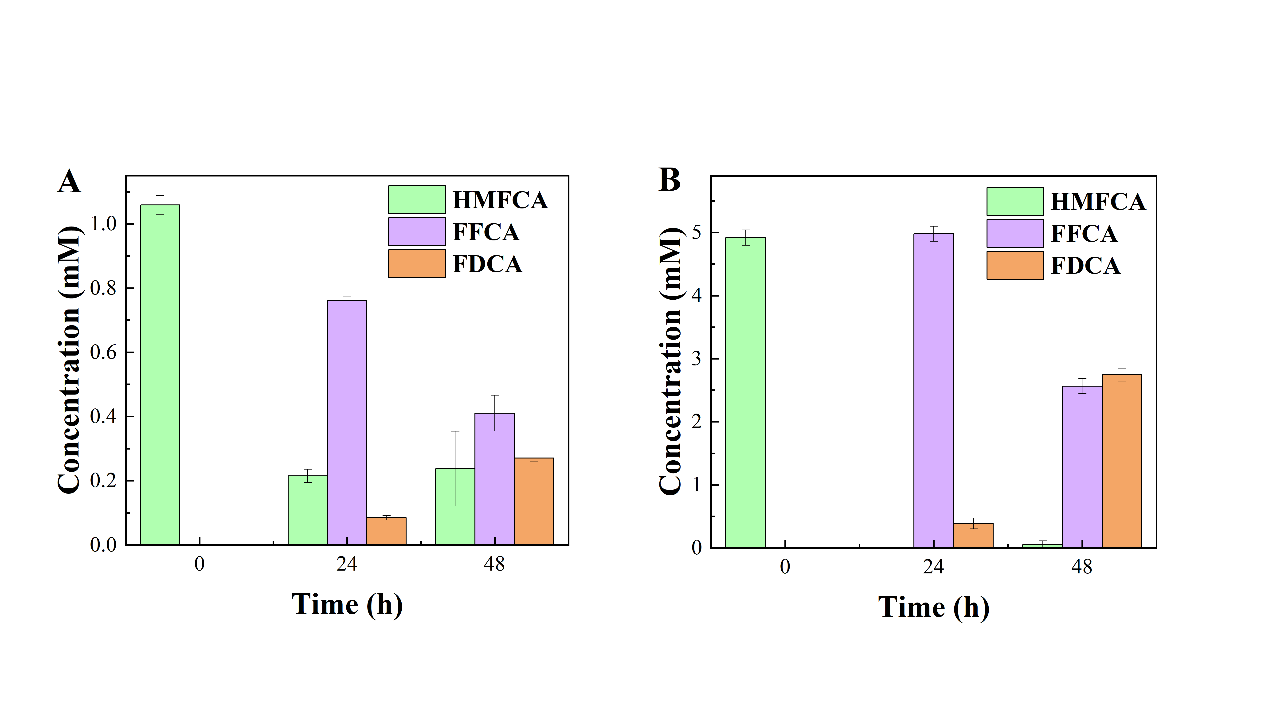


**Figure S3** Verification of the product inhibition on HMFCA oxidation by *Cgl*AlcOx through two individual experiments. (A) The reaction mixtures contain 1 mM HMFCA and 1 U/mL *Cgl*AlcOx. (B) The reaction mixtures contain 5 mM HMFCA and 5 U/mL *Cgl*AlcOx.

**Table S1** The chemical shifts of characteristic peaks of HMF and its derivatives.

| Compounds | Functional group | Chemical shifts (ppm) |
| --- | --- | --- |
| HMF | aldehyde group | 9.33 |
|  | furanic ring | 7.41, 6.61 |
|  | hydroxymethyl group | 4.58 |
| HMFCA | furanic ring | 7.17, 6.46 |
|  | hydroxymethyl | 4.54 |
| DFF* | unhydrated aldehyde | 9.33 |
|  | furanic ring | 7.38, 6.59 |
|  | hydrated aldehyde | 5.93 |
| DFF | aldehyde group | 9.59 |
|  | furanic ring | 7.43 |
| FFCA | furanic ring | 7.23, 7.46 |
|  | aldehyde | 9.5 |
| FDCA | furanic ring | 7.11 |

**Table S2** Summary of previously reported FDCA production from HMF via enzymatic cascade reactions.

| Enzymatic cascades | Substrate concentration (mM) | HMF conversion (%) | Yield of FDCA (%) | References |
| --- | --- | --- | --- | --- |
| Galactose oxidase/ alcohol dehydrogenases/HRP | 10 | > 99 | 95 | ^1^ |
| 5-Hydroxymethylfurfural oxidase/lipase | 6 | > 99 | 94 | ^2^ |
| Aryl-alcohol oxidase/unspecific peroxygenase | 2 | 100 | 100 | ^3^ |
| Aryl alcohol oxidase/peroxygenase/galactose oxidase | 10 | 100 | 80 | ^4^ |
| Mutated aryl-alcohol oxidase/catalase | 1.5 |  | 100 | ^5^ |
| Galactose oxidase/ periplasmic aldehyde oxidase/ catalase/ horseradish peroxidase | 100 |  | 100 | ^6^ |
| Immobilized laccase/ Novozym 435 (lipase)/TEMPO | 20 |  | 94 | ^7^ |
| Alcohol oxidases (*Cgl*AlcOx) /catalase/ horseradish peroxidase | 10 | 100 | 9 | ^8^ |
| Methanol oxidase/ Aryl-alcohol oxidase/ unspecific peroxygenase/methanol | 1.5 | 100 | 98 | ^9^ |
| Immobilized lipase B/ galactose oxidase/ horseradish peroxidase | 30 |  | 88 | ^10^ |
| Aryl-alcohol oxidase/ unspecific peroxygenase | 3 | 100 | 91 | ^11^ |

**References:**

1.Jia H-Y, Zong M-H, Zheng G-W, Li N. One-Pot Enzyme Cascade for Controlled Synthesis of Furancarboxylic Acids from 5-Hydroxymethylfurfural by H_2_O_2_ Internal Recycling. ChemSusChem. 2019;12(21):4764-8.

2.Wu S, Liu Q, Tan H, Zhang F, Yin H. A Novel 2,5-Furandicarboxylic Acid Biosynthesis Route from Biomass-Derived 5-Hydroxymethylfurfural Based on the Consecutive Enzyme Reactions. Applied Biochemistry and Biotechnology. 2020;191(4):1470-82.

3.Lappe A, Jankowski N, Albrecht A, Koschorreck K. Characterization of a thermotolerant aryl-alcohol oxidase from *Moesziomyces antarcticus* oxidizing 5-hydroxymethyl-2-furancarboxylic acid. Applied Microbiology and Biotechnology. 2021;105(21):8313-27.

4.Karich A, Kleeberg SB, Ullrich R, Hofrichter M. Enzymatic Preparation of 2,5-Furandicarboxylic Acid (FDCA)-A Substitute of Terephthalic Acid-By the Joined Action of Three Fungal Enzymes. Microorganisms. 2018 Jan 9;6(1):5.

5.Serrano A, Calviño E, Carro J, Sánchez-Ruiz MI, Cañada FJ, Martínez AT. Complete oxidation of hydroxymethylfurfural to furandicarboxylic acid by aryl-alcohol oxidase. Biotechnology for Biofuels. 2019;12(1):217.

6.McKenna SM, Mines P, Law P, Kovacs-Schreiner K, Birmingham WR, Turner NJ, et al. The continuous oxidation of HMF to FDCA and the immobilisation and stabilisation of periplasmic aldehyde oxidase (PaoABC). Green Chemistry. 2017;19(19):4660-5.

7.Chang X, Zhang C, Gao L, Liu X, You S, Qi W, et al. Tandem Biocatalysis by CotA-TJ102@UIO-66-NH_2_ and Novozym 435 for Highly Selective Transformation of HMF into FDCA. Transactions of Tianjin University. 2019;25(5):488-96.

8.Cleveland ME, Mathieu Y, Ribeaucourt D, Haon M, Mulyk P, Hein JE, et al. A survey of substrate specificity among Auxiliary Activity Family 5 copper radical oxidases Cellular and Molecular Life Sciences. 2021;78(24):8187-208.

9.Carro J, Fernández-Fueyo E, Fernández-Alonso C, Cañada J, Ullrich R, Hofrichter M, et al. Self-sustained enzymatic cascade for the production of 2,5-furandicarboxylic acid from 5-methoxymethylfurfural. Biotechnology for Biofuels. 2018;11(1):86.

10.Qin Y-Z, Li Y-M, Zong M-H, Wu H, Li N. Enzyme-catalyzed selective oxidation of 5-hydroxymethylfurfural (HMF) and separation of HMF and 2,5-diformylfuran using deep eutectic solvents. Green Chemistry. 2015;17(7):3718-22.

11.Carro J, Ferreira P, Rodríguez L, Prieto A, Serrano A, Balcells B, et al. 5-hydroxymethylfurfural conversion by fungal aryl-alcohol oxidase and unspecific peroxygenase. The FEBS Journal. 2015;282(16):3218-29.
